# Transcriptional program for nitrogen starvation-induced lipid accumulation in *Chlamydomonas reinhardtii*

**DOI:** 10.1186/s13068-015-0391-z

**Published:** 2015-12-02

**Authors:** Adrián López García de Lomana, Sascha Schäuble, Jacob Valenzuela, Saheed Imam, Warren Carter, Damla D. Bilgin, Christopher B. Yohn, Serdar Turkarslan, David J. Reiss, Mónica V. Orellana, Nathan D. Price, Nitin S. Baliga

**Affiliations:** Institute for Systems Biology, 401 Terry Ave N, Seattle, 98109 WA USA; Jena University Language and Information Engineering (JULIE) Lab, Friedrich-Schiller-University Jena, Jena, Germany; Research Group Theoretical Systems Biology, Friedrich-Schiller-University Jena, Jena, Germany; Sapphire Energy Inc., San Diego, CA USA; Polar Science Center, University of Washington, Seattle, WA USA; Departments of Bioengineering and Computer Science and Engineering, University of Washington, Seattle, WA USA; Departments of Biology and Microbiology, University of Washington, Seattle, WA USA; Molecular and Cellular Biology Program, University of Washington, Seattle, WA USA; Lawrence Berkeley National Lab, Berkeley, CA USA

**Keywords:** Network modeling, Phenotypic transition, Transcriptional regulatory network, Metabolic network, Lipid accumulation, *Chlamydomonas reinhardtii*

## Abstract

**Background:**

Algae accumulate lipids to endure different kinds of environmental stresses including macronutrient starvation. Although this response has been extensively studied, an in depth understanding of the transcriptional regulatory network (TRN) that controls the transition into lipid accumulation remains elusive. In this study, we used a systems biology approach to elucidate the transcriptional program that coordinates the nitrogen starvation-induced metabolic readjustments that drive lipid accumulation in *Chlamydomonas reinhardtii*.

**Results:**

We demonstrate that nitrogen starvation triggered differential regulation of 2147 transcripts, which were co-regulated in 215 distinct modules and temporally ordered as 31 transcriptional waves. An early-stage response was triggered within 12 min that initiated growth arrest through activation of key signaling pathways, while simultaneously preparing the intracellular environment for later stages by modulating transport processes and ubiquitin-mediated protein degradation. Subsequently, central metabolism and carbon fixation were remodeled to trigger the accumulation of triacylglycerols. Further analysis revealed that these waves of genome-wide transcriptional events were coordinated by a regulatory program orchestrated by at least 17 transcriptional regulators, many of which had not been previously implicated in this process. We demonstrate that the TRN coordinates transcriptional downregulation of 57 metabolic enzymes across a period of nearly 4 h to drive an increase in lipid content per unit biomass. Notably, this TRN appears to also drive lipid accumulation during sulfur starvation, while phosphorus starvation induces a different regulatory program. The TRN model described here is available as a community-wide web-resource at http://networks.systemsbiology.net/chlamy-portal.

**Conclusions:**

In this work, we have uncovered a comprehensive mechanistic model of the TRN controlling the transition from N starvation to lipid accumulation. The program coordinates sequentially ordered transcriptional waves that simultaneously arrest growth and lead to lipid accumulation. This study has generated predictive tools that will aid in devising strategies for the rational manipulation of regulatory and metabolic networks for better biofuel and biomass production.

**Electronic supplementary material:**

The online version of this article (doi:10.1186/s13068-015-0391-z) contains supplementary material, which is available to authorized users.

## Background

Green algae hold great promise for the manufacture of renewable biofuels [1]. The relatively low yield of algae-based biofuels, however, does not currently make them an economically viable replacement for fossil fuels [1]. Several strategies have been used to improve biofuel production, from strain design to improvements in growth, harvesting and refining techniques [2], but a large gap still needs to be bridged. Green algae accumulate lipids when subjected to nutrient depletion [3–10] and other stresses [11–13]. In parallel, nutrient starvation also causes growth arrest, thus limiting biomass accumulation, which is a major disadvantage for large-scale biofuel production. Consequently, an obvious strategy for the improvement of microalgae biofuel yield would be the decoupling of lipid accumulation from growth arrest at the molecular level. Such a rational strain design strategy requires a comprehensive mechanistic understanding of the transcriptional regulatory network (TRN) controlling lipid accumulation in order to identify the key regulatory elements (e.g., transcription factors (TFs) or metabolic bottlenecks), which coordinate the transition from nutrient starvation to growth arrest and lipid accumulation.

Our understanding of lipid accumulation in green algae has recently benefited from the use of high-throughput technologies to track genome-wide transcriptional changes during state transitions. In this regard, *C. reinhardtii* has emerged as the *de facto* model organism for algal biofuels research [12]. The early analyses of whole transcriptome changes in *C. reinhardtii* [6] uncovered the main hallmarks of nitrogen (N) starvation and lipid accumulation, i.e. the downregulation of protein synthesis and photosynthetic apparatus and redirection of primary carbon (C) metabolism. Subsequent high-resolution time series transcriptome experiments revealed that the TF nitrogen-responsive regulator-1 (NRR1) is in part responsible for the transcriptional changes that result in lipid accumulation during N starvation [14]. More integrative views of the phenotype transition during N starvation [15, 16] highlighted the importance of early transcriptional responses and gradual upregulation of alternative pathways of N assimilation and C metabolism. In addition to transcriptomic approaches, quantitative proteomic methods have provided unique insights into the global metabolic adjustments that drive N starvation-induced lipid accumulation, uncovering complex activity changes in the enzymes responsible for C and N metabolism [17–19]. For instance, proteomics analysis of lipid bodies revealed lipid accumulation to be a complex process involving lipid synthesis and recycling, as well as lipid trafficking and signaling to maintain homeostasis in microalgae oil bodies [20]. Further comprehensive analysis integrating transcriptome and proteome measurements revealed the multilevel responses of N-sparing mechanisms [21]. Metabolomics studies have also been conducted to characterize *C. reinhardtii* response to N starvation, which revealed differential flux signatures under N deprivation [22–24]. Finally, other macronutrient starvation studies, such as for sulfur (S) and phosphorus (P), have allowed for comparative transcriptome analyses. While several characteristic physiological changes are shared across N, S and P starvations (e.g., photosynthesis apparatus downregulation, reduced C fixation and lipid accumulation), other acclimation responses are nutrient-specific, such as the upstream controllers of the starvation response signaling circuit (e.g., SNRK2.1 or PSR1 [25, 26]), triacylglycerol (TAG) accumulation temporal profiles [27] and thylakoid membrane conservation [28].

The availability of high-throughput data sets provides an opportunity to use computational modeling approaches to obtain added insight into the process of lipid accumulation. In particular, the computational inference of TRNs from genome-wide gene expression datasets should be achievable, as a suite of methods are available to construct such network models [29–31]. For instance, the cMonkey algorithm [32], a semi-supervised biclustering algorithm that uses gene expression data guided by biologically informative priors and *de novocis*-acting gene regulatory element (GRE) detection, has been successfully applied to numerous organisms across all domains of life to build accurate and predictive models of TRNs [33–38]. High-throughput data sets also provide an opportunity to study the behavior of metabolism at a systems level. In this regard, constraint-based modeling approaches such as flux balance analysis [39] have proved to be particularly valuable for genome-scale investigation of metabolic flux distributions and for the development of metabolic engineering strategies in many species [40–45] while they can also serve as valuable platforms for data integration [46].

To obtain mechanistic insight into the transcriptional response of *C. reinhardtii* to N starvation, we employed a systems-level approach to unravel the program responsible for coordinating transcriptional changes that occur from the onset of N starvation to lipid accumulation. Through the integration of multiple publicly available data sets and resources, we used cMonkey to build the first mechanistic TRN model for the transition from N starvation into lipid accumulation in *C. reinhardtii*. To assess the impact of transcriptional regulation affecting metabolism, we integrated the TRN model with a genome-scale metabolic model for *C. reinhardtii* [47]. This enabled prediction of metabolic flux distributions during N starvation, as well as the identification of putative targets for increasing lipid yield. Furthermore, we conducted a comparative analysis of the expression pattern of key metabolic genes between N, S and P starvation experiments to identify common and condition-specific responses to those macronutrient starvations. Thus, through the integration of co-regulated modules identified from the time course analysis into a comprehensive metabolic model of *C. reinhardtii*, we obtained a systems-level understanding of how genome-wide transcriptional changes induced by N starvation drive the metabolic shift into a lipid accumulation state.

## Results and discussion

**Fig. 1:**
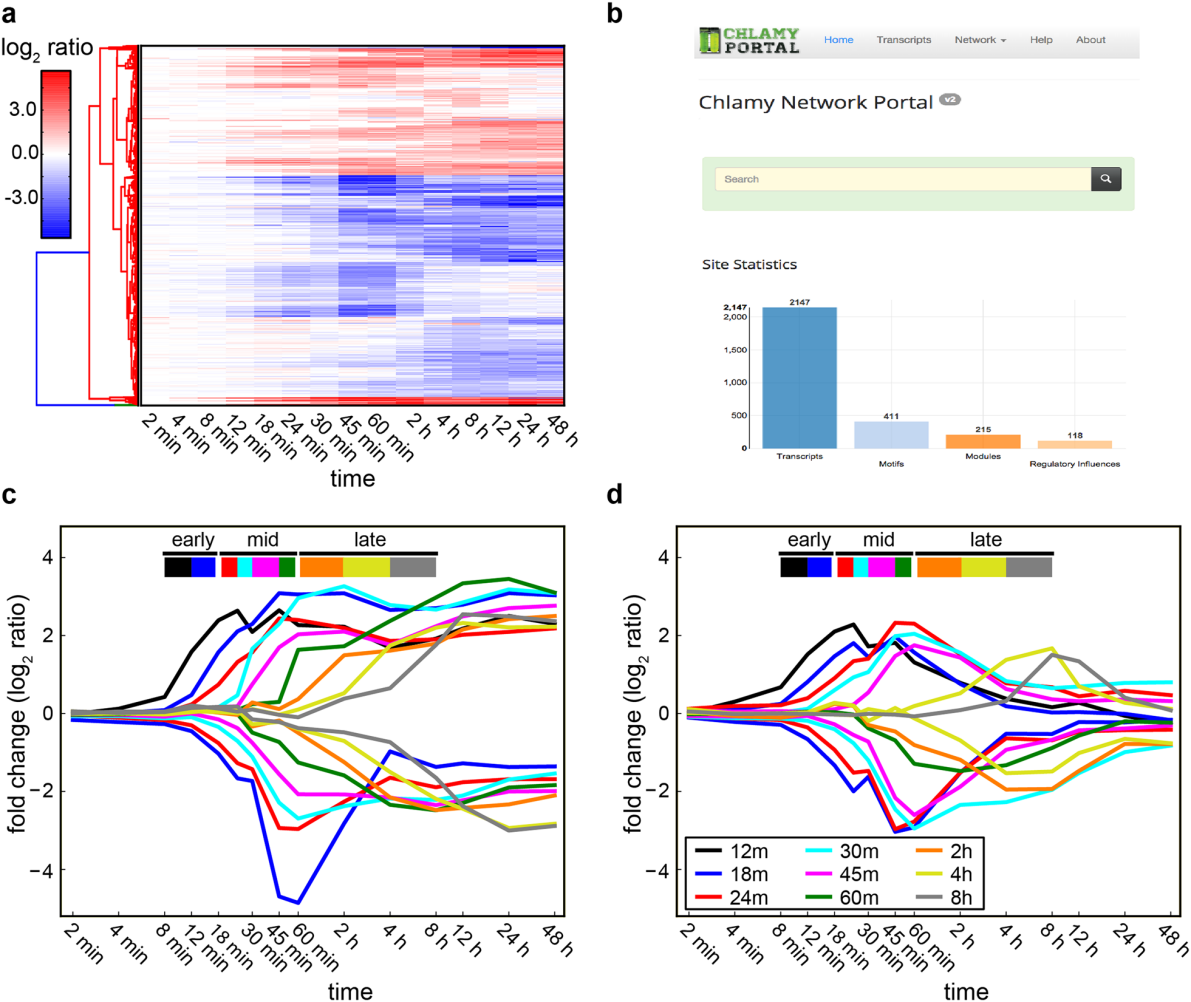
Transcriptional response of *C. reinhardtii* to N starvation and lipid accumulation. **a** Heatmap representation for the hierarchical clustering of the log_2_ expression changes in 2,147 post-filtered set of transcripts. *Red* (*blue*) indicates a relative increase (decrease) of expression. Color intensities are proportional to fold change magnitude. **b** Default view of the front page of the Chlamy Network Portal. In the portal, 2,147 transcripts are organized into 215 modules, 118 regulatory influences and 411 motifs. The site includes a powerful Apache Solr-based faceted search and navigation tools. The portal content is also linked to other information resources like Phytozome, STRING, GO categories and relevant literature. **c**, **d** Differentially expressed transcripts organized as a sequential set of transcriptional waves: 17 monotonic transcriptional waves composed of 125 transcriptional modules and 1,482 transcripts (**c**), and 15 transient transcriptional waves of 55 transcriptional modules and 758 transcripts (**d**). Each *line* represents average fold change for a given transcriptional wave. The timestamp on each wave reflects the timepoint at which transcript level change crosses a twofold threshold

To infer a N starvation-specific TRN model for *C. reinhardtii*, we integrated complementary data from: (1) the *C. reinhardtii* genome annotation [48] (version 5.5, available from Phytozome [49]), (2) the highest resolution time series transcriptome data set available for *C. reinhardtii* N starvation [14], and (3) a network of functional protein–protein interactions [50]. Analysis of the publicly available transcriptome data resulted in the identification of a set of 2,147 transcripts that showed significant difference in abundance between the onset of N starvation and lipid accumulation (see “Methods”; Fig. 1a). This set of transcripts constitutes the core network of transcriptionally regulated genes responsible for sensing N starvation and orchestrating the subsequent physiological shift to the lipid accumulation phenotype. We analyzed this set of 2147 transcripts with cMonkey [32] and identified a total of 215 putatively co-regulated modules, which were organized into a high confidence TRN model that can be explored through the interactive Chlamy Network Portal [51] (Fig. 1b).

The 215 co-regulated transcriptional modules were combined based on their temporal profiles to generate an ordered sequence of 31 transcriptional waves of two types: *monotonic*, in which transcript abundances changed and achieved new steady state levels; and *transient*, in which transcript abundances changed transiently before returning to the pre-starvation levels (Fig. 1c, d, Additional file 1: Table S1). The sizes of these transcriptional waves ranged from 17 to 340 transcripts, while their temporal schedules ranged from 12 min (min), for the earliest responding transcriptional modules, through 8 h (h) for late-stage responses. We labeled each wave by the timestamp at which the mean gene expression level of the corresponding transcriptional module(s) crossed the twofold threshold relative to the pre-starvation condition. The overall schedule of changes can be divided into three major stages of transcriptome transitions: *early*-*stage response* from 0 to 18 min, which was characterized by changes in transcripts for N transport, cellular signaling, ionic composition and protein translation; *mid*-*stage response* from 18 to 60 min, marked by the reorganization of metabolism; and *late*-*stage transition* between 1 and 8 h just before cells undergo a phenotypic state transition marked by lipid accumulation. We describe below a temporally organized summary of changes, with selected highlights of major events that occur at each of the three stages of the N starvation response (Table 1 serves as a reference for summary and highlights, and Additional file 1: Table S1 contains a comprehensive list of all transcripts associated with each stage, wave and module).

**Table 1:** Summary of the organization of the transcriptional changes

Stage	Time	Trend	Dynamics	Waves, modules, transcripts	Functional highlights (corrected *p* value)
Early	0–18 min	Up	Monotonic	2, 7, 105	Ammonium transport (1.7e−9) Potassium ion transmembrane transport (1.4e−5) Ion channel activity (2.8e−3) Ion transport (3.4e−3) Protein kinase activity (1.3e−2)
			Transient	2, 7, 118	
		Down	Monotonic	1, 1, 17	Ribosome biogenesis (6.3e−8) Transcription (3.7e−6) rRNA processing (2.9e−5) Pseudouridine synthesis (7.0e−5) Methyltransferase activity (9.0e−5)
			Transient	1, 17, 202	
Mid	18–60 min	Up	Monotonic	4, 24, 304	Mitochondrial pyruvate transport (1.0e−6) Glutamine biosynthetic process (3.0e−5) Lipid metabolic process (5.2e−5) Amine metabolic process (7.5e−5) Proteolysis (9.6e−3)
			Transient	3, 7, 110	
		Down	Monotonic	4, 44, 552	Purine nucleotide biosynthetic process (5.1e−5) Fatty acid beta-oxidation (4.9e−4) Pseudouridine synthesis (8.7e−4) Malate dehydrogenase activity (1.6e−3) Fatty acid biosynthetic process (1.9e−3)
			Transient	4, 15, 237	
Late	1–8 h	Up	Monotonic	3, 12, 177	DNA replication (8.6e−7) Microtubule cytoskeleton organization (1.1e−4) NADP binding (3.5e−3) Lipid metabolic process (1.8e−2) Nucleotide binding (2.4e−2)
			Transient	2, 8, 139	
		Down	Monotonic	3, 47, 489	Photosynthesis (7.7e−31) Protein folding (1.0e−13) Fructose-bisphosphate aldolase activity (8.4e−6) Cell redox homeostasis (4.9e−5) Fatty acid biosynthetic process (7.5e−3)
			Transient	2, 5, 95	

Transcriptional transition is organized within three time stages according to their temporal schedules. Transcripts are organized into co-regulated modules, which in turn are compiled into transcriptional waves. Selected significantly enriched functional categories (GO terms) are shown

### The early-stage response: sensing a new environment

The early-stage response occurred from 0 to 18 min, with the earliest consistent transcriptional change (>twofold) initiating at 12 min. This stage included 442 transcripts in 32 co-regulated modules that were organized into 6 waves –122 transcripts were in 3 waves that changed monotonically (105 upregulated (2 waves), 17 downregulated (1 wave)), while 320 transcripts were in 3 waves that changed transiently [118 upregulated (2 waves), 202 downregulated (1 wave)] (Table 1, Additional file 1: Table S1). As expected, the earliest transcriptional changes were directly related to the N starvation response and included up to an 8-fold upregulation of 4 ammonium transporters and permeases (*AMT3*-*5* and *AMT8*). In addition, transcripts encoding the mitochondrial carbonic anhydrase *CAH5* and the ABC transporter *HLA3*, key metabolic proteins involved in the regulation of the C/N ratio homeostasis [52, 53], were among the earliest changing metabolic transcripts. The early-stage response also appeared to be devoted to modulating intracellular ionic composition through the upregulation of 12 membrane ion transporters, including Ca^2+^, Na^+^, K^+^, and Zn^2+^ transporters. Nineteen transcripts associated with signaling and modulation of growth and stress response were also upregulated, including transcripts of the mitogen-activated protein kinase (MAPK) pathway (e.g., *PP2C*, *STK24*, *MAPK7*), brassinosteroids biosynthesis pathway (e.g., *DET2* [54]), and the auxin pathway (e.g., Ran-binding protein 9 (Cre01.g007150) [55]). Transcripts encoding proteins involved in protein degradation were also upregulated at this stage including at least 10 transcripts associated with the ERAD/ubiquitination pathway (e.g., *CDC48* [56] and Cre01.g038950, a Sep15 domain protein [57]). Overall, 92 % of the transcriptional repression in the early-stage occurred transiently and mainly impacted ribosome biogenesis and RNA processing proteins, including RNA methylation (Supplementary Table S1). These early-stage transcriptional changes likely set the stage for the major metabolic restructuring that occurs during the mid-stage response.

### Mid-stage response: metabolic state transition

The mid-stage response was comprised of 1203 differentially regulated transcripts within 90 co-regulated modules. These modules were organized into 15 waves that changed between 24 and 60 min after N starvation (Fig. 1c, d). Of these transcripts, 856 changed monotonically (304 were upregulated (4 waves), and 552 were downregulated (4 waves)) and 347 changed transiently (110 upregulated (3 waves), 237 downregulated (4 waves)). A significant fraction of the mid-stage transcriptional response was devoted to metabolic N scavenging, as evidenced by the upregulation of 14 transcripts encoding key metabolic enzymes involved in N scavenging from amino acids, nucleotides and polyamines (Table 2, Additional file 2: Table S2). Concomitantly, 12 transcripts responsible for the key steps of purine, pyrimidine, amino acids and polyamines biosynthesis were all downregulated (Additional file 2: Table S2). Targeted ubiquitin-mediated protein degradation during the early-stage response might feed amino acids into these N-scavenging and recycling processes. Consistent with reduced biosynthesis of amino acids, 41 transcripts encoding components of the protein synthesis and folding machinery were also downregulated (Table 2, Additional file 2: Table S2). Collectively these changes serve to mitigate the consequences of N starvation by conserving internal N resources for cellular sustenance upon growth arrest. Furthermore, the downregulation of the chaperones may indicate a higher rate of protein misfolding [58], and as a consequence higher endoplasmic reticulum stress which has been directly associated with the induction of lipid bodies [59].

**Table 2:** Transcriptional changes on the cellular hallmarks of N starvation

Trend	Pathway	Representative examples	Timestamp
Up	Pentose phosphate pathway	Glucose-6-phosphate dehydrogenase	18 min
	Purine catabolism	Xanthine dehydrogenase Adenosine deaminase	18–30 min
	Urea metabolism	Carbamoyl phosphate synthetase Ornithine transcarbamylase Agmatine deiminase Allophanate hydrolase Urea carboxylase	24–30 min
	Glutamine metabolism	Glutamine synthetase	30 min
	Polyamines oxidation	Copper amine oxidase	18 min–2 h
	TAG biosynthesis	Diacylglycerol acyltransferase	24–45 min
Down	FA β-oxidation	Acyl-CoA oxidases 2,4 dienoyl-CoA reductase	24 min
	Purine and pyrimidine biosynthesis	Adenylosuccinate synthase Uridine 5′-monophosphate synthase	24 min
	FA biosynthesis	3-ketoacyl-CoA-synthase Biotin synthase Acetyl-CoA carboxylase, 3-ketoacyl-ACP reductase	24–45 min
	Translation	Translation initiation factors Translation elongation factors Release factors	24 min–4 h
	Amino acid biosynthesis	Threonine deaminase Shikimate dehydrogenase Dihydrodipicolinate synthase 3-Phosphoglycerate dehydrogenase	24–30 min
	Protein folding	Chaperonins	45 min
	Calvin cycle	Triose phosphate isomerase Transketolase Ribose-5-phosphate isomerase	45 min
	Carbon concentrating mechanism	Malate dehydrogenase	45 min
	Polyamines biosynthesis	Ornithine decarboxylase Adenosylmethionine decarboxylase Spermidine synthase	45 min–4 h
	Glycerolipid metabolism	Phosphatidate cytidylyltransferase Glycerol-3-phosphate acyltransferase	45 min–4 h
	Chlorophyll biosynthesis	Chlorophyll synthase Geranylgeranyl diphosphate synthase Geranylgeranyl reductase Uroporphyrinogen decarboxylase	60 min–4 h
	Two-component peroxide-detoxifying system	NADPH-dependent thioredoxin reductase 2-Cys peroxiredoxin Peroxidase	2 h
	Photosynthesis	Ferredoxin Photosystem I and II proteins ATP synthase Cytochrome b_6_f complex subunits PsbP	2–8 h
	Glutathione-ascorbate cycle	Ascorbate peroxidase Prohibitin, thioredoxin	4 h
	Glycolysis	Glyceraldehyde-3-phosphate Dehydrogenase	4 h

Summary of key cellular pathways differentially regulated with representative transcript examples. Timestamp and direction of regulation (trend) are indicated

Transcripts encoding proteins involved in many carbon sequestration processes were downregulated during the mid-stage response including those implicated in carbon concentrating mechanisms and those encoding several key enzymes of the Calvin cycle (5 transcripts in 10 co-regulated modules in 1 wave, see Additional file 2: Table S2). Other C metabolism enzymes that were downregulated include glyceraldehyde-3-phosphate dehydrogenase (Cre01.g010900), which has been recently implicated in lipid accumulation in *Arabidopsis* [60] and the periplasmic carbonic anhydrase 1 (*CAH1*), which exhibited a dramatic drop in abundance from a maximum level of ~1500 to 0 FPKM (fragments per kilobase of transcript per million mapped reads). The overall repression of C fixation is an indication of transition into a lowered metabolic state as a consequence of the cessation of growth. In addition, alternative sources of carbon such as recycled C-backbones from degraded protein and acetate influx might also compensate for the downregulation of C fixation.

While lipids start to accumulate at 8 h after N starvation [14], within 60 min post N starvation we observed the upregulation of transcripts encoding enzymes responsible for maintaining the cellular redox balance (high NADPH/NADP^+^) required for lipid biosynthesis [61] and synthesis of lipid precursor metabolites (e.g., *GLD2* [62]). Similarly and as previously described by Boyle et al. [14], transcripts for TAG biosynthesis *DGAT1* and *DGTT1* were upregulated. Importantly, *DGAT1*, which catalyzes the rate-limiting step in the *de novo* TAG biosynthesis, increased up to fourfold, 45 min after N starvation. Membrane remodeling [63] was enhanced through the upregulation of membrane lipases such as saposin (Cre05.g235700), indicative of catabolism of glycosphingolipids, which could facilitate membrane remodeling and in extreme cases membrane lysis [64]. Notably, transcripts encoding key enzymes for the *de novo* fatty acid (FA) biosynthesis, such as the three β-ketoacyl-(acyl-carrier-protein) synthases (*KAS1*, *KAS2* and *KAS3*; Cre11.g467723, Cre07.g335300 and Cre04.g216950, respectively), which are responsible for the first enzymatic step catalyzed by the multi-enzyme complex fatty acid synthase (FAS) were all downregulated. Importantly, the downregulation of lipid biosynthesis transcripts continued through the late-stage of the N starvation response.

### Late-stage response: transition into the lipid accumulation state

**Fig. 2:**
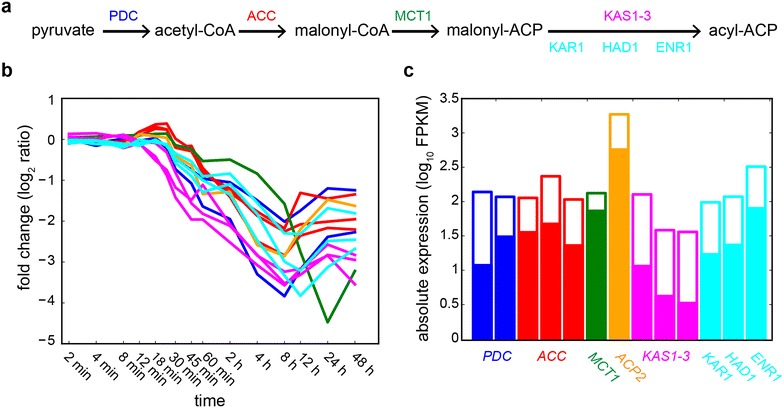
FA biosynthesis pathway is transcriptionally downregulated during N starvation. **a** Schematic view of the reaction steps for FA biosynthesis and the enzymes that catalyze them. **b** Expression profile for transcripts encoding enzymes highlighted in (**a**). **c** Absolute expression values for FA biosynthesis transcripts in log_10_ FPKM (*open bars* represent absolute expression level at time t = 0 while solid bars represent levels at t = 8 h). *PDC* pyruvate dehydrogenase complex, *ACC* acetyl-CoA carboxylase, *MCT1* malonyl-CoA:ACP transacylase, *ACP2* acyl carrier protein, *KAS* β-ketoacyl-(ACP) synthase, *KAR1* 3-ketoacyl-ACP reductase, *HAD1* β-hydroxyacyl-ACP dehydratase, *ENR1* enoyl-ACP-reductase

The observed commencement of significant lipid accumulation 8 h after N starvation was set up by transcriptional changes occurring in the late-stage response, which began after 1 h from N starvation. Comprised of 665 monotonic (177 up-, 489 downregulated) and 234 transient (139 up-, 95 downregulated) transcripts organized into 72 co-regulated modules in 10 waves, the late-stage response was characterized by lipid metabolism remodeling, downregulation of photosynthesis, oxidative stress and protein folding stress responses, and an upregulation of sugar catabolism. While an increased metabolic flux into FA biosynthesis would be expected to support TAG production, the late-stage response involved the downregulation of many transcripts associated with FA biosynthesis such as the chloroplastic pyruvate dehydrogenase complex (PDC), acetyl-CoA carboxylase (ACC), and malonyl-CoA:ACP transacylase, all involved in the biosynthesis of FA precursors (Fig. 2a, b). Interestingly, upregulation of some of these enzymes such as PDC is known to be strongly correlated with lipid accumulation [65]. Additionally, the downregulation of FA biosynthesis-related transcripts during the mid-stage response (i.e., downregulation of *KAS1*-*3*), continued into the late-stage response with the transcriptional downregulation of the 3 enzymes of the FAS complex involved in the subsequent steps of FA biosynthesis: 3-ketoacyl-ACP reductase (Cre03.g172000), β-hydroxyacyl-ACP dehydratase (Cre03.g208050) and enoyl-ACP-reductase (Cre06.g294950). The FAs 16:0 and 18:1 are the most abundant of the TAG fractions in N starved cells [28], reflecting the importance of *de novo* FA synthesis for TAG accumulation, which accounts for 79 % of the TAG content in N starved cells [63], providing further evidence that the lipid accumulation response is a time-dependent additive process. Indeed, transcriptional downregulation of FA biosynthesis enzymes during lipid accumulation is consistent with previous observations in *C. reinhardtii* [14, 21, 23] and other microalgae [66, 67], but not in diatoms [68, 69]. Downregulation at the transcriptional level is consistent with a strong reduction in protein levels for enzymes of the early steps of the FA biosynthesis, particularly ACC [21, 70]. It is of note that although FA biosynthesis enzymes were transcriptionally downregulated, their absolute abundance remained relatively high at this stage, ranging from a minimum value of 3 FPKM for *KAS3* to 572 FPKM for *ACP2*, presumably to maintain flux towards FA biosynthesis (Fig. 2c). Furthermore, while transcript abundance provides insights into changes in metabolic activity, actual enzyme levels can deviate from transcript levels based on other factors such as protein stability and turnover. Therefore, long-term lipid accumulation might be accomplished by increasing the overall carbon flux towards the FA pathway [71] or, as previously noted, by limiting the catabolism of lipids through the FA β-oxidation pathway during lipid accumulation [72]. Besides, several FA desaturases (*FAB2*, *FAD3*, *FAD7*), which incorporate double bonds into FAs at the cost of reducing equivalents, were downregulated, potentially to conserve energy while producing lower amounts of unsaturated FAs.

The transcript encoding the mitochondrial glycerol-3-phosphate dehydrogenase (*GPD2*, Cre01.g053000), which serves as a major link between carbohydrate metabolism and lipid metabolism, is upregulated as part of the late-stage response, in agreement with previous studies of the *C. reinhardtii* transcriptome during N starvation [18, 21]. Interestingly, *Phaeodactylum tricornutum* cells overexpressing *GPD* had a 60 % increase in neutral lipid content, reaching 39.7 % of dry cell weight in stationary phase [73], plus overexpression of *GPD* has also been reported to increase lipid content in plants [74]. By contrast, the cytoplasmic isoform *GPD1* (Cre12.g511150) was downregulated as part of a late-stage response, highlighting the importance of cellular compartmentalization to achieve modularity in metabolism. Additionally, consistent with the previously described role for lipases in lipid accumulation [6], the TAG lipase expression profiles changed, with *TGL9* and Cre09.g391838 upregulated, while *TGL15* was downregulated up to 18-fold. Undoubtedly, TAG lipases are key modulators of TAG accumulation in *C. reinhardtii* as previous works have demonstrated [75, 76]. The extensive transcriptional changes in genes encoding lipid composition enzymes underscore the importance of regulatory mechanisms for the overall lipid increase and changed lipid composition during N starvation in *C. reinhardtii.*

Other pathways associated with biosynthesis of membrane components that were downregulated in the late-stage response (see Table 2, Additional file 2: Table S2) included biosynthesis of glycerolipids and isoprenoids (at least 7 key enzymes), porphyrin and chlorophyll (at least 4 key enzymes). Downregulation of the photosynthetic function and apparatus is a major hallmark of N starvation [77]. Our model accounts for the entire chloroplast light reaction chain, including photosystem I and II, cytochrome b_6_f, plastocyanin, ferredoxin, ferredoxin-NADP^+^ reductase and ATP synthase, comprising 54 transcripts distributed in 35 co-regulated modules and 4 waves (see Table 2, Additional file 2: Table S2). This downregulation during the late-stage response likely results in lower production of reactive oxygen species, and is consistent with the observation that many oxidative stress response systems were also downregulated (at least 9 transcripts of key oxidative protection enzymes).

### The TRN for the N starvation response

**Fig. 3:**
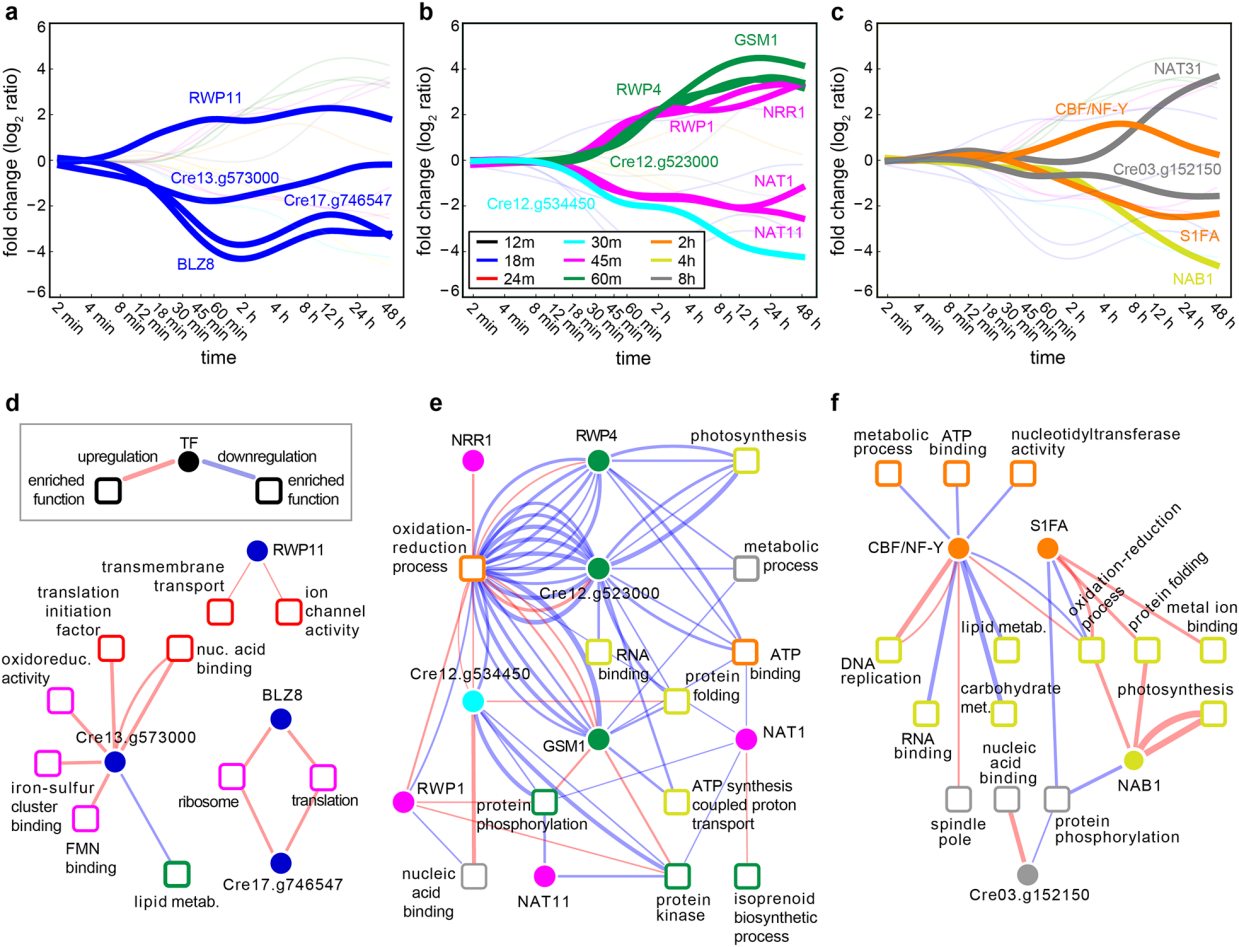
Expression dynamics and network of predicted transcriptional influences. **a**–**c** *Top panels* show TR expression dynamics: each profile is labeled with the relevant TR it represents. **d**–**f** Network of predicted transcriptional influences from TRs (*circle nodes*) on enriched GO terms (*square nodes*) through transcriptional modules (edges). Edge *color* indicates transcriptional activation (*red*) or repression (*blue*) influenced of a TR on the transcriptional module. Edge thickness is inversely proportional to the distance between the TR and the transcriptional module. Nodes are *colored* using the same pattern as in Fig. 1c, d, e.g., *blue* represents a differential expression acquired at time 18 min after N starvation

In order to construct the network of transcriptional regulators (i.e., TFs and chromatin regulators) that coordinate the N starvation response, we computed the distances between temporally ordered, smoothed expression profiles of the transcriptional regulators (TRs) and the transcriptionally co-regulated modules. We selected the first fifth percentile of all distances within the physiologically relevant window of 15–90 min time lag between a change in the TR and its putative target transcriptional module. This time window was selected based on the time it takes for a differentially regulated TR to exert an observable effect on transcription ([75]; see “Methods” for details). Despite the possible contribution of post-transcriptional regulatory events in modulating transcriptional activity, this analysis uncovered a TRN in which 17 of the 34 differentially regulated TRs exerted 118 putative TR-module regulatory influences, coordinating 815 transcripts within 60 co-regulated modules (Table 3, Additional file 3: Table S3). This TRN model encompasses 17 waves across the three stages of the N starvation response (Fig. 3).

**Table 3:** List of the TRs predicted to orchestrate the transcriptional response during N starvation

Stage	Name	Description	Timestamp (min)	Trend	Predicted regulated modules
Early	Cre13.g573000	SET domain methyltransferase	12	Down	40, 113, 162, 197, 127
	BLZ8	Basic region leucine zipper	13	Down	105, 121
	RWP11	RWP-RK TF	15	Up	150
	Cre17.g746547	bZIP TF	15	Down	105, 121
Mid	CGL86	Nuclear inhibitor of protein phosphatase-1	26	Down	129, 130, 99, 172, 13, 45, 82, 148
	NAT11	Acetyltransferase (GNAT)	30	Down	129
	RWP1	RWP-RK domain protein	36	Up	130, 99, 13, 45, 82, 148
	NAT1	Acetyltransferase (GNAT)	37	Down	200, 178, 93, 174
	NRR1	SBP domain	44	Up	82, 67
	RWP4	RWP-RK domain-containing protein	48	Up	193, 98, 35, 37, 72, 170, 45, 142, 175, 59, 99, 53, 87, 27, 95
	GSM1	Gamete-specific minus 1	50	Up	193, 98, 35, 37, 166, 172, 45, 175, 99, 59, 67, 53, 54, 87, 27, 149
	Cre12.g523000	Zinc finger CCCH domain containing protein	62	Up	149, 193, 98, 35, 37, 102, 170, 172, 45, 142, 175, 99, 59, 67, 53, 54, 87, 185, 27, 211, 95
Late	CGL107	Histone-like transcription factor (CBF/NF-Y)	109	Up	193, 8, 9, 141, 50, 24, 154, 59, 92, 158
	Cre09.g386753	DNA binding protein S1FA	109	Down	98, 35, 166, 172, 175, 67, 149, 27
	NAB1	Nucleic acid binding protein	146	Down	33, 34, 133, 104, 76, 144, 136, 210, 21, 151, 57, 186
	NAT31	Acetyltransferase (GNAT)	371	Up	104, 136, 133
	Cre03.g152150	Zinc finger C2H2 type domain	418	Down	129, 13

Seventeen differentially expressed TRs during N starvation have a predicted significant influence on at least one transcriptional module. TR name and description, first time point of differential expression (timestamp) and predicted influenced modules are indicated

Based on this TRN model, the transcriptional cascade started within 12 min after N starvation with the twofold upregulation of a previously described N response regulator in *C. reinhardtii* and plants, *RWP11* [78]. By 18 min, three other TRs were downregulated: *BLZ8* and Cre17.g746547, both bZIP TFs, and Cre13.g573000, a SET domain methyltransferase (Fig. 3a, d). The subsequent remodeling of metabolism during the mid- and late-stage response was coordinated by at least 8 TRs that acted from 24 min to 2 h after N starvation. One of these TRs is *NRR1*, the squamosa promoter binding protein described by Boyle et al. [14] which reaches twofold upregulation 44 min after N starvation (Fig. 3c, e). Interestingly, *RWP1*, a RWP-RK domain TF [79], showed a very similar temporal expression pattern as *NRR1*. In the 48–68 min time frame, three other TFs crossed the twofold upregulation threshold: Cre12.g523000 (a C3H Zinc finger TF), *RWP4* and *GSM1*. While *GSM1* is known to function as a regulator of the minus mating type gametogenesis response, the function of these other two TFs is less well understood. Additionally, other TRs downregulated at this point include a FHA domain nuclear inhibitor of protein phosphatase-1 (Cre12.g534450) and several acetyltransferases (*NAT1* and *NAT11*). The late transcriptional wave was putatively orchestrated by the transient upregulation of the subunit D of NF-Y TF (Cre07.g341800) [80], the transcriptional downregulation of the DNA binding protein S1FA [81], and the C2H2-family TF Cre03.g152150 (Fig. 3c, f). Other changes in TRs during the late-stage transcriptional waves include the upregulation of the *NAT31* acetyltransferase and the downregulation of *NAB1* (Cre06.g268600), which plays an important role in controlling the expression of the light-harvesting antenna of photosystem II in *C. reinhardtii* [82]. Finally, we found 17 additional transcriptional regulators with differential expression during the phenotype transition, but their expression profile did not match significantly any of the transcriptional modules, suggesting a more cooperative mode of action involving multiple TRs regulating the same transcriptional module. Additional file 4: Fig. S1 shows the dynamics of such TRs, with notable examples including the early downregulation of the AP2-family TF Cre06.g275500, up to 33-fold increase of the bHLH TF Cre01.g011150 during the mid-stage response and the upregulation of *RWP8* during the late stage. While incomplete, the TRN model organized the complex set of transcriptional changes during the N starvation response into a modular framework of co-regulated transcripts that were temporally coordinated by as few as 34 TRs, allowing for model-based systematic analysis of metabolic consequences.

### Metabolic network analysis

**Fig. 4:**
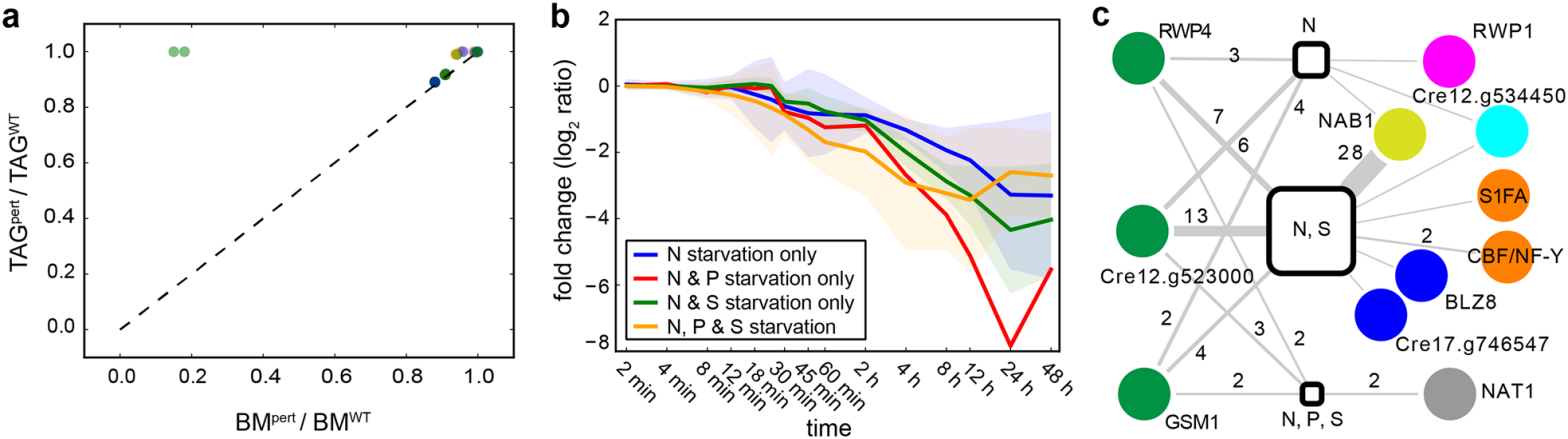
Metabolic targets for higher relative TAG to biomass ratio. **a** Model-predicted consequences of transcriptional downregulation on relative TAG per unit biomass. *Dots* represent prediction for 57 metabolic enzymes whose downregulation resolves in increased TAG per unit biomass (above the diagonal, ρ > 1). Among these 57 metabolic targets, only 10 genes (*blue dots*) are uniquely downregulated in N starvation, and 40 genes are also downregulated during S starvation (*green dots*). Five genes are downregulated in N, S and P (*orange dots*) and just one, Cre02.g082750, is downregulated in N and P only (*red dot*). **b** Average expression profiles during N starvation for the metabolic targets grouped as in **a**. **c** Network of transcriptional regulatory influences on metabolic targets. Each *circle* node represents a TR (*colored* as in Fig. 3). Edges represent the predicted regulatory influence of a given TR on specific genes across the different starvation responses (*squared nodes*). Edge labels indicate the number of genes regulated by each TR, one if no label is present

Using the available genome-scale metabolic network of *C. reinhardtii* [47], we performed a variation of flux balance analysis to assess the impact of knocking down the metabolic flux through reactions catalyzed by enzymes that were transcriptionally downregulated within specific co-regulated modules. In order to estimate the TAG content per biomass, we computed a dimensionless ratio (ρ) of relative TAG production per biomass accumulation, which allowed us to predict the impact of knocking down a transcript on the amount of lipids per unit biomass (see “Methods” for details). From the 1,058 monotonically downregulated transcripts, this approach led to the prediction that the knockdown (KD) of 57 transcripts would result in a relative increase of TAG production per biomass with respect to wild type conditions (i.e., ρ > 1) (Fig. 4a, Additional file 5: Table S4). Investigation of the expression patterns for these 57 transcripts revealed that they were not uniformly distributed along the whole time domain of transcriptional changes, but were mainly confined to the 30 min to 4 h range, coinciding with the metabolic state transition (Fig. 4b, Additional file 5: Table S4). The 57 transcripts were distributed across 51 modules and this TRN mapping further implicated guilt-by-association of 671 additional genes in driving lipid accumulation. The 12 TRs of these modules are predicted by the TRN to be putatively responsible for coordinating down regulation of these modules with other transcriptional changes during the N-starvation response. Notable functions represented among these modules (protein synthesis, signaling and photosynthesis) suggest a potential mechanistic linkage between transcriptional programs for growth arrest, the photosynthetic machinery and lipid accumulation during N-starvation (Fig. 4c).

Metabolic targets changing earliest fall into the ATP homeostasis category, particularly control of ATP/NADPH ratios, like the nucleoside-diphosphate kinase (Cre07.g325734), the cytochrome b_6_f complex (responsible for creating the proton gradient that drives the synthesis of ATP in chloroplasts) and plastocyanin (Cre03.g182551), which is responsible for the flow of electrons between cytochrome b_6_f and photosystem I. Furthermore, we identified several targets from central metabolism like triose phosphate isomerase (TPI). TPI catalyzes the reversible conversion of dihydroxyacetone phosphate (DHAP) to glyceraldehyde-3-phosphate (GA3P) and coordinates many pathways including glycolysis, Calvin cycle, and glycerol metabolism. Notably, recent studies in *Arabidopsis* have uncovered the crucial role of TPI in general lipid metabolism [83]. Indeed, a TPI mutant in *Arabidopsis* with a fivefold reduction in transcript levels accumulated DHAP and glycerol, both byproducts of TAG mobilization and precursors for glycerolipid biosynthesis. Another key target identified from this analysis was Cre02.g080200, which encodes for the transketolase TRK1 which is an essential enzyme of both the Calvin cycle and the non-oxidative pentose phosphate pathway (PPP). TRK1 has also been found to play a key role in the regulation of C allocation through calcium-dependent phosphorylation regulation [84]. Our metabolic modeling approach also predicted that the KD of the next enzyme of the non-oxidative PPP, ribulose phosphate-3-epimerase (Cre12.g511900) results in a ρ > 1. The metabolic targets predicted to affect TAG production during the late-stage response between 2 and 4 h after N starvation were strongly dominated by genes involved in photosynthesis. Subunits of photosystem I and II, including light-harvesting proteins, chlorophyll binding proteins, thylakoid lumen proteins and ferredoxins, were all predicted to result in a ρ > 1 upon KD, in agreement with cell growth arrest and recycling of the photosynthetic apparatus. Interestingly, other metabolic targets downregulated in the 2–4 h period whose KD resulted in a ρ > 1, were involved in lipid biosynthesis functions, including enzymes like acyl-carrier-protein dehydratase (Cre03.g208050), farnesyl pyrophosphate synthase (Cre03.g207700) or geranylgeranyl diphosphate reductase (Cre01.g050950), which may be responsible of the observed changes in lipid composition in *C. reinhardtii* during N starvation. These observations may reflect both network redundancy on TAG production and how different categories of lipids affect biomass, as defined in the metabolic model biomass function.

Finally, we used the model to identify the metabolic genes predicted to be involved in the biosynthesis of acetyl-CoA, malonyl-CoA and glycerol 3-phosphate, key precursors for lipid biosynthesis. Out of the 100 genes predicted to be involved in this process, 26 are downregulated and only four are transcriptionally upregulated (Additional file 6: Table S5). The upregulated genes include glycerol-3-phosphate dehydrogenase and glyceraldehyde 3-phosphate dehydrogenase, key enzymes for the FA biosynthesis [73, 85], involved in the production of glycerol and reducing equivalents, respectively. The 26 downregulated enzymes participate at different levels of FA biosynthesis, ranging from carbon fixation and the acetyl-CoA and malonyl-CoA synthesis to CoA biosynthesis (Additional file 6: Table S5). Furthermore, we compared the pattern of expression of these metabolic targets under other nutrient starvations, e.g., S and P [25]. Virtually all the metabolic targets were also downregulated in S starvation conditions. We observed, however, a remarkably different expression pattern in P starvation, where the vast majority of these metabolic targets were not downregulated (Additional file 6: Table S5, Fig. 4c). Indeed, FA composition of accumulated TAG differs from N to P starvation, specially in 16:0 and 18:2 fractions [28], reflecting different routes of TAG production. These observations evidence how the metabolic and regulatory changes that occur during lipid accumulation in response to N and S starvation are similar, while P starvation induces a different lipid accumulation program.

## Conclusion

Analysis of expression dynamics at the whole transcriptome scale for *C. reinhardtii* has uncovered a complex but structured transcriptional response during N starvation and subsequent transition into a state of lipid accumulation. The application of the biclustering algorithm cMonkey revealed that the transcriptional response was modular and organized as 31 temporally ordered waves that were triggered as early as 12 min and continued changing until 8 h after N starvation. Based on the timing and functional composition of the waves the N starvation response could be divided into three categories: the early response characterized by distinct cellular signaling, the mid response recapitulating how the cell metabolism transitions into a different state and the late response with hallmarks of the acquisition of the final phenotype, dominated by transcripts for photosynthesis, lipid metabolism, oxidative stress and protein folding. Furthermore, we predicted a network of transcriptional regulators, at least 17 TFs and chromatin remodeling proteins that putatively orchestrate the transcriptional response. Certainly, the phenotypic transition is not the consequence of regulation by a single TF, but the coordinated response of several factors. In fact, integration of the TRN model with a genome-scale metabolic flux model demonstrated that there are 57 metabolic bottlenecks that are distributed across different modules of the transcriptional program. The TRN mediates the coordinated downregulation of these 57 metabolic steps to drive increased lipid per unit biomass. Similar expression patterns of the 57 enzymes under S starvation are suggesting that the same TRN might oversee both the S- and N-starvation responses. In contrast, different metabolic and transcriptional networks appear to be responsible for the P-starvation response, wherein only 6 of the 57 enzymes were differentially expressed. Ultimately, to ensure the integration of the TRN model with other existing resources and broader dissemination among the scientific community, we developed an accessible web-based resource, the Chlamy Network Portal [51], which incorporates the TRN model together with the processed expression data and offers filtering and visualization interfaces for further analysis. Well-known resources such as Phytozome [49], STRING [50], and Gene Ontology (GO) terms [86] are seamlessly integrated at the Chlamy Network Portal.

Recent studies in *C. reinhardtii* [21, 70, 79] and other microalgae [87] have integrated various omics data sets to reveal that cellular response to N starvation is controlled by the structure of a TRN, and cannot be explained by individual action of a handful of TRs. Schmollinger et al. [21] performed a very comprehensive study of the cellular response to N starvation at different regulatory levels, quantifying the transcriptome, proteome and metabolome for both wild type and mutant strains. They uncovered several N-sparing mechanisms and listed a set of differentially regulated TFs as candidate regulators of the cellular response. In order to identify the key regulatory genes involved in the control of lipid metabolism, Gargouri et al. [79] used a time-lagged correlation analysis to identify putative TRs of cellular metabolism, before and after the onset of lipid accumulation. Our approach is distinct in that it integrates different types of evidence for co-regulation to infer a mechanistic and predictive TRN. Furthermore, by integrating this model with a metabolic model we were able to predict consequences of specific transcriptional changes on biomass production as well as metabolic flux towards TAG accumulation. The modular architecture of this TRN predicts TR regulation of not single transcripts or metabolites, but rather co-regulated modules enabling a robust assessment of the functional consequences of TR changes. Importantly, Valledor et al. [70] combined transcriptomics, proteome and metabolome measurements during N starvation and repletion to demonstrate how such integrative approaches reveal new understandings of molecular regulation. A beautiful example is the discovery of a central role for the major lipid droplet protein within such regulatory and metabolic network model in linking signaling cascades (GTPases downregulated during N starvation) to vesicle formation (COP II) and lipid body formation.

There are several possible limitations to our study. First, our TRN is based on expression data and transcriptional changes do not fully account for changes at the protein level, much less for functional activity of post-transcriptionally regulated proteins [88, 89], which may result in phenotypic changes not recapitulated by the transcriptional response. Therefore, integration of proteome and metabolome measurements would represent a powerful complement to our approach. At the level of the integration of the metabolic and TRN models, potential missing links may occur from the incomplete nature of the currently available metabolic models for *C. reinhardtii* consisting on 1080 genes and 2190 reactions, which is far from the complete metabolome set. Indeed, in a parallel effort, our group recently extended and improved the metabolic model for *C. reinhardtii*, upgrading it to 1355 genes (1460 transcripts), 2394 reactions and 1133 metabolites, enabling very accurate predictions of gene deletion growth phenotypes, with an area under the receiver operating characteristic curve of 0.92 [90]. Also, the lack of an exhaustive annotation for the TF set is another source for missing interactions between potential regulators of the transcriptional modules. Additionally, other potential regulators of gene expression like miRNAs are not currently included in our TRN model. Finally, the experimental validation of *de novo* detected GREs and predicted TF to transcriptional module interactions would produce a more accurate picture of the functional interactions recapitulated by our TRN.

We foresee two main future directions of this study: first, the expansion of the TRN model with expression data from other phenotypic transitions, e.g., starvation on other nutrients, different growing conditions (light, trophic conditions, CO_2_ levels, etc.), and different mutant strains. A second direction would be to refine the accuracy and specificity of the transcriptional regulatory influences with the integration of quantitative data that includes additional influencers, like miRNAs [91–95] or post-translational modifications [96]. Moreover, a genome-wide chromatin state map would constitute a resourceful informational tool for detecting and associating GREs to the transcriptional response [97, 98]. Recent experimental evolution work with *C. reinhardtii* [99–101] has shown the adaptive plasticity of this organism to quickly generate new phenotypes with increased fitness to novel environments. Experimental evolution approaches and gene editing [102] would be complementary and a good synergistic combination to TRN modeling to better understand the process of lipid accumulation in microalgae, with the ultimate goal of engineering new strains with desired phenotypes.

Given the complexity underlying the phenotypic transitions in *C. reinhardtii*, we are confident that the constructed TRN model presented here represents a relevant predictive tool that would uniquely guide the rational selection of candidate gene targets for improved biofuel production. The dissemination of the TRN through the Chlamy Network Portal platform will especially ensure an efficient broadcast of the model to the growing biofuel research community.

## Methods

### Expression data

Raw RNA-Seq expression data obtained from a N starvation time course experiment on *C. reinhardtii* wild type CC-3269 [14] was aligned with STAR [103] to the current genome annotation (version 5.5 from Phytozome [49]). Next, using cuffdiff [104], we computed for each transcript *i* at each time point *t*, the log_2_ expression ratio, $r_{i}^{t}$,

1 $$r_{i}^{t} = \log_{2} \left( {(x_{i}^{t} + 1)}/{(x_{i}^{t = 0} + 1)} \right)$$

where $x_{i}^{t}$ represents the expression in fragments per kilobase of transcript per million mapped reads (FPKM) of transcript *i* at time *t*. We filtered out unchanging transcripts and noisy expression measurements by selecting any transcript that complied with any of the following two rules: (1) $r_{i}^{t} > {\text{abs}}\left( 1 \right)$ during the lipid accumulation time points, i.e., at time points 8 h, 12 h, 24 h and 48 h after N starvation [14] or (2) $r_{i}^{t} > {\text{abs}}\left( 1 \right)$ consistent for a single period of at least 30 min.

### TRN inference

We used cMonkey [32] version 4.9.11 to build the TRN model. We set the searching and scanning window as (−10,2000) base pairs upstream from the transcriptional start site of common *cis*-acting gene regulatory elements (GREs). We obtained the TRs annotation from Schmollinger et al. [21], which contained 491 unique transcriptional regulator genes for the current version of *C. reinhardtii* annotation. Protein–protein functional interaction network was retrieved from STRING [50], version v9.05.

### Inference of transcriptional regulators influences

Transcript expression trajectories were first smoothed with the csaps routine from MATLAB. Smoothed data was then divided by its maximum absolute value to account for non-linear relationships between transcriptional regulators and transcriptional modules. A matrix of distances between TRs and transcriptional modules was calculated allowing for a defined best time lag between the expression of the TR and the corresponding transcriptional module. For eukaryotic genes, it can take from several minutes up to several hours since the expression of a TF changes, to transcriptional changes on its target genes [105]. We used a time window of 15–90 min as time lag, based on reported values in literature [106]. Specifically, we allowed first for 5–20 min for the nuclear phase: transcription, transport and export (Ref. [107] and Bionumbers BNID:105650). As for the transcription time, we allowed 3–8 min; such time window is supported by the ~3 min mean translation time previously reported in yeast [108] in conjunction with additional data about translation rates of 5 to 11 aa/sec (Bionumbers BNID:104598 and BNID:109527, respectively [109, 110]). Then, we included 10 additional min into the time lag for protein translocation to the nucleus and binding to promoter sequences (BNID:109955), which sets our time lag ideally into the expected range of 18–38 min. Still, we searched for TR to transcriptional module interactions as late as 90 min, allowing for the effect of other factors like long sequences, protein maturation and other post-transcriptional regulatory mechanisms. We predicted as true TR to transcriptional module influences those whose best distance was smaller than the first fifth percentile of all the distances and also had a time delay within the defined time window of 15–90 min.

### Metabolic analysis of genome-scale metabolic model

We used the genome-scale metabolic reconstruction *i*RC1080, the hitherto most comprehensive metabolic model of *C. reinhardtii* [47]. We simulated mixotrophic growth by allowing for the uptake of acetate as well as light via warm and cool fluorescent light sources (reactions 4 and 5 of the model) to mimic the experimental setup of Boyle et al. [14]. N is available only via ammonium uptake, whereas starch uptake was prohibited. Consequently, the lower bounds of the respective exchange reactions were either left at their published values or set to zero (to prohibit uptake). To separate TAG production from the biomass production, we subtracted all TAG producing compounds from the mixotrophic biomass definition and created a separate exchange reaction EX_TAG with the same TAG composition as in the biomass definition and a new biomass reaction BM_TAG. Without further flux bound perturbations we termed this model iRC1080WT. We conducted multiple Flux Balance Analysis (FBA) [39] runs to investigate the influence of reaction or module perturbation on TAG production in a dimensionless form. We first computed the FBA with BM_TAG as objective function from iRC1080_WT to derive the unperturbed biomass flux BM_TAG_WT. To avoid numerical problems, we considered a KD as a reduction of the flux to at least 1/16th of the original flux. based on the particular reaction flux from the flux distribution that generates BM_TAG_WT. Cell viability was ascertained by requiring at least 10 % biomass production with respect to the original wild type predictions. We again conducted an FBA to derive the BM_TAG_pert from the perturbed network iRC1080_pert. The ratio BM_TAG_pert/BM_TAG_WT provides then the relative change of BM_TAG flux. Next, we computed two further FBAs, this time with EX_TAG as objective function for iRC1080_WT and iRC1080_pert to derive TAG_WT and TAG_pert, respectively. The ratio TAG_pert/TAG_WT consequently provides the relative change in TAG production upon gene and affected reaction perturbation (taking the value of one for no change). Finally, we computed a ratio of these ratios in the following form:

2 $$\rho = \left( {{\text{TAG}}\_{\text{pert}}/{\text{TAG}}\_{\text{WT}}} \right)/\left( {{\text{BM}}\_{\text{TAG}}\_{\text{pert}}/{\text{BM}}\_{\text{TAG}}\_{\text{WT}}} \right)$$

The interpretation of Eq. 2 is thus straightforward: if* ρ* > 1 the model perturbation affects the biomass production more severely and causes, thus, more TAG content per biomass. Consequently, a value smaller than one relates to a perturbation that causes less TAG per biomass content. Note that Eq. 2 is dimensionless, because units cancel out in the TAG and BM ratios.

## Additional files

10.1186/s40064-015-1541-2 Comprehensive list of the transcriptional changes. As in Table 1, each transcriptional stage is hierarchically itemized into transcriptional waves, co-regulated modules and finally transcripts. Each wave has a link to a different sheet where each of the transcripts are defined, including their co-regulated module membership. Timestamp of co-regulated module is highlighted with different colors, using the same color map as in Fig. 1c-d.
10.1186/s40064-015-1541-2 Transcriptional changes on the cellular hallmarks of N starvation. For each functional hallmark, each differentially regulated transcript is grouped into a distinctive pathway. Phytozome locus name, gene names and description, co-regulated module membership, timestamp and direction of regulation are indicated.
10.1186/s40064-015-1541-2 List of the TRs predicted to orchestrate the transcriptional response during N starvation. Seventeen differentially expressed TRs during N starvation with a predicted significant influence on at least one transcriptional module. TR annotation (locus name, gene name and description), first time point of differential expression, predicted influenced modules and the enriched functions of those modules are indicated. Numbers in parenthesis indicate the number of network-predicted metabolic targets included within each module.
10.1186/s40064-015-1541-2 Expression dynamics for additional TRs. Each panel shows the expression dynamics for the early (**a**), mid (**b**) and late (**c**) responses of the TRs for which no significant match to a co-regulated module was found.
10.1186/s40064-015-1541-2 List of predicted genes with ρ > 1. List of transcripts downregulated during N starvation predicted to result in higher TAG to biomass ratio upon gene knockdown. For each gene, we present the computed ρ value, the co-regulated module membership, the time of module downregulation and the predicted TR(s) regulating such module(s).
10.1186/s40064-015-1541-2 List of metabolic TAG production related genes varying during N starvation and LAP. At least 30 metabolic genes that have a direct functional role in TAG production are differentially expressed during N starvation. Expression pattern of these metabolic targets in S starvation is much more similar than in P starved cells. N starvation co-regulated module membership and predicted TRs are also shown.

## Abbreviations

GRE: gene regulatory element
TRN: transcriptional regulatory network
KD: knockdown
TAG: triacylglycerol
FA: fatty acid
TR: transcriptional regulator
TF: transcription factor
