# Supplementary figures and images for: Transcriptional program for nitrogen starvation-induced lipid accumulation in Chlamydomonas reinhardtii
Source: Biotechnol Biofuels. 2015 Dec 2;8:207. doi: 10.1186/s13068-015-0391-z (PMC4667458; doi:10.1186/s13068-015-0391-z)

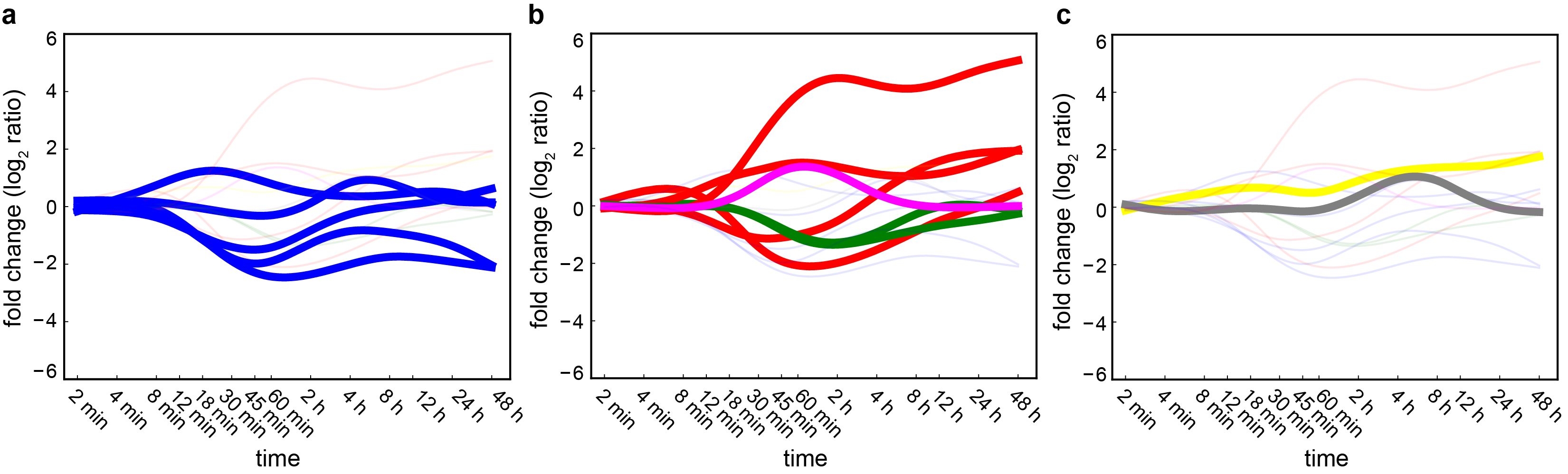

Supplement: Supplementary file 4 — 10.1186/s40064-015-1541-2 Expression dynamics for additional TRs. Each panel shows the expression dynamics for the early (a), mid (b) and late (c) responses of the TRs for which no significant match to a co-regulated module was found. [file 13068_2015_391_MOESM4_ESM.png]
